# Neural Network-Based Strong Motion Prediction for On-Site Earthquake Early Warning

**DOI:** 10.3390/s22030704

**Published:** 2022-01-18

**Authors:** You-Jing Chiang, Tai-Lin Chin, Da-Yi Chen

**Affiliations:** 1 Department of Computer Science and Information Engineering, National Taiwan University of Science and Technology, Taipei 106335, Taiwan; M10815085@mail.ntust.edu.tw; 2 Seismological Center of Central Weather Bureau, Taipei 100006, Taiwan; dayi@cwb.gov.tw; 3 Department of Earth and Life Sciences, University of Taipei, Taipei 100234, Taiwan

**Keywords:** ground motion prediction, earthquake early warning, neural network

## Abstract

Developing on-site earthquake early warning systems has been a challenging problem because of time limitations and the amount of information that can be collected before the warning needs to be issued. A potential solution that could prevent severe disasters is to predict the potential strong motion using the initial P-wave signal and provide warnings before serious ground shaking starts. In practice, the accuracy of prediction is the most critical issue for earthquake early warning systems. Traditional methods use certain criteria, selected through intuition or experience, to make the prediction. However, the criteria thresholds are difficult to select and may significantly affect the prediction accuracy. This paper investigates methods based on artificial intelligence for predicting the greatest earthquake ground motion early, when the P-wave arrives at seismograph stations. A neural network model is built to make the predictions using a small window of the initial P-wave acceleration signal. The model is trained by seismic waves collected from 1991 to 2019 in Taiwan and is evaluated by events in 2020 and 2021. From these evaluations, the proposed scheme significantly outperforms the threshold-based method in terms of its accuracy and average leading time.

## 1. Introduction

Destructive earthquakes often lead to significant loss of life and property. Issuing warnings before the arrival of strong waves is a potential way to reduce this loss during disasters. Regional earthquake early warning (EEW) systems usually detect seismic waves near the epicenter and provide warnings to more remote regions [1,2]. However, the blind zone close to the epicenter may suffer from severe shaking. Many studies have therefore investigated methods for providing on-site warnings [3,4,5]. In contrast to the traditional methods, this paper investigates the methods based on ground motion prediction using artificial intelligence techniques.

The characteristics in the initial part of P-wave have been used for various prediction purposes. A number of studies have been dedicated to revealing the relationship between the magnitude of an earthquake and the characteristics of its initial P-wave [6,7]. Wu and Zhao [8] used the peak ground displacement (Pd) from the initial P-wave to estimate the magnitude, and showed that Pd can be an indicator for estimating ground motion. The methods of Wu [3] and Zollo [4] used Pd and the period parameter $\tau_{c}$ of the initial portion of the P-wave to predict ground motion. Both methods provided warnings with a decent leading time before the beginning of strong shaking. However, the difficulty of selecting the thresholds for the checking criteria may easily result in false or missed alarms. In addition, the criteria used in these methods are usually selected through intuition or experience and may not capture certain information representative of the rupture. In contrast, the artificial intelligence techniques can extract significant features from the raw input data and predict the potential results for the investigated targets. The accuracy of the prediction is envisioned to be improved.

In this study, an intelligent strong motion prediction (ISMP) method is proposed to predict earthquake ground shaking for on-site earthquake early warning. The proposed scheme uses a convolutional neural network (CNN) in an attempt to obtain a relationship between the characteristics extracted from the initial P-wave and the strong motion. The input data used are the three-axis wave acceleration signals and do not require preprocessing. Compared with the traditional methods, this technique can more effectively capture the implied features from multi-axis data, which may not even be observed by experienced seismologists. ISMP is intended to predict whether the maximum peak ground acceleration (PGA) of the tested seismic waves exceeds a pre-defined threshold using the first few seconds of the P-wave signal. The models were trained and tested using seismic waves collected from the Taiwan Strong Motion Instrumentation Program (TSMIP) network from 1991 to 2019 and the Central Weather Bureau Seismic Network (CWBSN) from 2014 to 2019. Experiments were also conducted to evaluate the performance of the proposed scheme using different durations of the initial P-wave time window. Based on the results, the proposed scheme outperforms the traditional threshold-based method in terms of accuracy and average leading time. Sample cases from 2020 and 2021 were selected from the CWBSN to evaluate the performance of the proposed scheme and illustrate the superiority against the traditional threshold-based methods.

The rest of the paper is organized as follows. Section 2 addresses related work. Section 3 presents the proposed system models for early ground motion prediction. Section 4 presents the datasets and the performance evaluation of the proposed scheme. Finally, conclusions are provided in Section 5.

## 2. Related Work

Ground motion prediction is a crucial issue for on-site EEW systems. Traditional on-site EEW methods usually use earthquake parameters measured from the original signal to trigger the system. Wu et al. [3] proposed a threshold-based method to predict strong ground shakings. If Pd is greater than a pre-selected threshold within a short initial time window of the P-wave, a warning is triggered. The results showed that using the criterion of Pd > 0.5 cm within the first three seconds after the P-wave arrival can predict the PGA of the subsequent destructive seismic waves. The approach was improved by Hsieh et al. [9], who analyzed four inland earthquakes recorded by a P-wave alert device network and showed that it provided a highly successful detection rate with no false alarms.

In California, Böse et al. [10] proposed a new trigger criterion using two earthquake parameters, namely Pd and the period parameter $\tau_{c}$ from the first three seconds of the P-wave record. The scheme significantly reduced the number of false alarms in seven local earthquakes in 2007. In 2014, Böse et al. [11] applied the previous scheme to develop a complete system, named the California Integrated Seismic Network ShakeAlert. In Italy, Caruso et al. [12] also used the two parameters, $\tau_{c}$ and Pd, to rapidly estimate the earthquake intensity for on-site EEW. The scheme showed a high percentage of successful intensity predictions. In Taiwan, the method of Hsieh [9] is currently used in a high-density seismic network. It provides a sufficient leading time and a highly successful detection rate.

In recent years, artificial intelligence techniques have been applied to predict the ground motions at seismic stations [13,14]. Böse et al. [13] used artificial neural networks to estimate the peak ground velocity (PGV), epicentral distance, and magnitude of earthquakes. The results showed that the peak acceleration is significant in discriminating between an earthquake and noise, while the peak velocity and the peak displacement are relevant for estimating the magnitude and PGV. Hsu et al. [14] used support vector regression (SVR) to predict the PGA using six features extracted from the initial vertical component of the P-wave. These features are the peak acceleration, the peak velocity, the peak displacement, the effective predominant period, the cumulative absolute velocity, and the integral of the square velocity. The results showed that the SVR approach using these features extracted from the first three seconds can generate a smaller standard deviation of the predicted PGA from earthquake records collected between 1992 and 2006. The scheme can thus predict the approximate value of the real PGA.

CNN has been applied to several seismic problems, such as earthquake detection [15], earthquake localization [16], earthquake arrival-time picking [17,18], and PGA prediction [19,20]. The above studies [15,16,17,18,19,20] show that CNN techniques can extract implicit features from the waveforms to obtain useful information. For PGA prediction, Jozinović et al. [19] presented a CNN-based model to predict the intensity of ground shaking. The scheme provides a reliable estimation of ground shaking 15 to 20 s after an earthquake occurs. Münchmeyer et al. [20] developed a transformer earthquake alerting model to predict the PGA in target areas. The model issues warnings by predicting whether the output probability of the PGA exceeds a pre-defined probability or not. The scheme achieves high alert performance and appropriate warning time in datasets from Japan and Italy.

## 3. System Model

**Figure 1:**
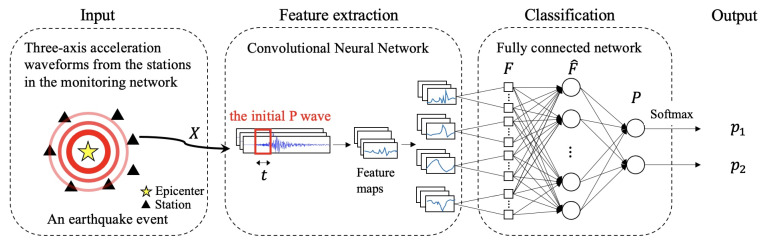
The proposed Intelligent Strong Motion Prediction (ISMP) model.

The proposed scheme intends to make a prediction for whether the PGA of an earthquake at a certain seismograph station will be greater than a pre-selected threshold η based on a small time window of the signal obtained when the P-wave arrives at the station. The proposed network consists of four components, i.e., input, feature extraction, classification, and output as shown in Figure 1. In the first stage, a number of seismic waves from the seismic monitoring networks are collected as the input. Secondly, the feature extraction component attempts to extract the relevant features and generate feature maps from the input waveforms. Then, the classification component categorizes the waveforms into output classes based on the extracted features. The details of these components and the training objective are described in this section.

### 3.1. Input

The input data are the three-axis acceleration sampling values in a small window of the initial P-wave. Suppose that *t* is the size of the window starting at the arrival of the P-wave. The input is a two-dimensional matrix $X\in R^{3\times t}$ containing three rows. Specifically, the matrix *X* can be defined as ${\left(x_{ij}\right)}_{1\leq i\leq 3,1\leq j\leq t}$, where $x_{ij}$ is the *j*th sample of the acceleration from the axis *i* in the initial window of the P-wave. Note that the values of the samples are the acceleration with the unit of $m/s^{2}$ rather than the raw counting values from the instruments.

### 3.2. Feature Extraction

For feature extraction, CNN is used to determine the relevant features for intensity prediction from the input data. The first step in the process uses one-dimensional convolution (Conv1D), which applies variant filters on the input data X to extract effective features from the time series. To boost the convergence speed and prevent the vanishing gradient problem, batch normalization [21] and rectified linear units [22] are added after every Conv1D layer. The batch normalization layer improves the stability of the training process and accelerates training speed, while rectified linear units are used to learn nonlinear and higher precision features for the activation function. Two Conv1D layers, which have 32 and 64 filters, are used in the scheme. All filter sizes are set to 3 and step sizes are set to 2.

After the convolution layer process, feature maps are generated. Each feature map represents a different abstraction of the seismic waves. Some may not have physical meaning and may be difficult to be identified by human beings. However, the CNN technique can find regularities in many waveforms to extract useful features.

### 3.3. Classification

In this component of the network, the learned feature maps from the previous layer are flatted to a one-dimensional vector $F\in R^{1\times n}$ and a feed-forward network is used to induce the decisions. Let *n* be the size of the vector *F* and *h* be the dimension of the fully connected layer. The feed-forward network aggregates the extracted features to obtain the global information as follows:

(1) $$\hat{F}=FW_{1},$$

where $W_{1}\in R^{n\times h}$ is the weight matrix tuned by the training process. Finally, the decision indicators for the two target classes, i.e., PGA ≥η and PGA <η, are generated as follows:

(2) $$D=\hat{F}W_{2},$$

where $W_{2}\in R^{h\times 2}$ is the weight matrix tuned by the training process.

### 3.4. Output

The model outputs the probabilities of the two classes, i.e., PGA ≥η and PGA <η. The softmax function is exploited to obtain the probability for each class. Specifically, the softmax function is given by:

(3) $$p_{i}=\frac{\exp(d_{i})}{\exp\left(d_{1}\right)+\exp\left(d_{2}\right)},$$

where $d_{i}$ is the decision indicator of the *i*th class and i∈{1,2}. In our case, $p_{1}$ is used as the probability of PGA ≥ η and $p_{2}$ as the probability of PGA < η. The scheme predicts that PGA will be greater than η if $p_{1}\geq p_{2}$; otherwise, the scheme predicts that PGA will be less than η.

### 3.5. Loss Function

The cross-entropy loss function applied in this study comprises the softmax function and the negative log-likelihood. Let $p_{k}^{j}$ denote the probability of the *k*th class for the *j*th waveform record as defined in Equation (3). The loss function can be expressed as:

(4) $$L=-\frac{1}{N}\sum_{j=1}^{N}\log\left(\max\{p_{1}^{j},p_{2}^{j}\}\right),$$

where *N* is the number of waveform records. Note that, by taking the logarithm, the larger the output probability $p_{k}^{j}$, the closer to zero the value of the loss.

In the training procedure, the optimizer AdamW [23] is adopted to minimize the loss and update the weight parameters. In addition, the learning rate decays as the number of training epochs increases. For each epoch, the learning rate will be multiplied by a factor of 0.9 to the learning rate used in the previous epoch.

## 4. Performance Evaluations

In this section, details of the dataset and the performance evaluations are described. In the experiments, the data from different time windows of the initial P-wave were selected as the input of the model to evaluate the performance of the proposed scheme on the testing set. The target magnitude threshold η used in the experiments was set to 80 Gal because seismologists have determined that most people experience panic during seismic waves of 80 Gal. All of the models were implemented by PyTorch [24]. The models were compared to the traditional threshold-based method (the Pd method) [9].

### 4.1. Dataset

**Figure 2:**
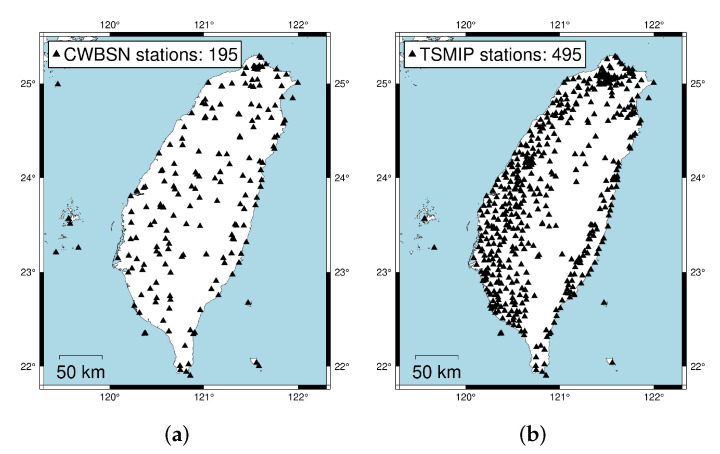
The figures show the station deployment of the seismological monitoring networks used in this work. (**a**) The station deployment of the Central Weather Bureau Seismic Network (CWBSN). (**b**) The station deployment of the Taiwan Strong Motion Instrumentation Program (TSMIP) network.

**Figure 3:**
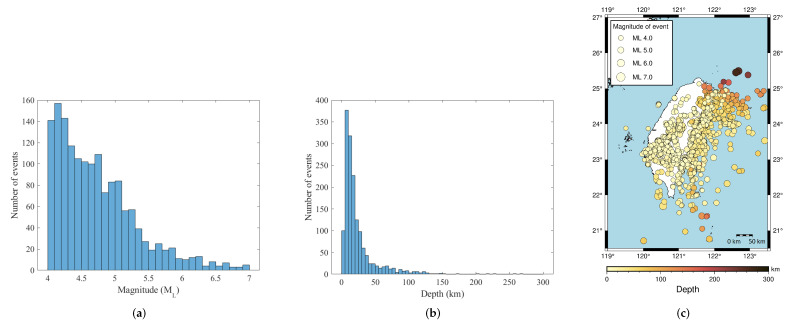
The statistics of the dataset. (**a**) The magnitude histogram. (**b**) The depth histogram. (**c**) The event locations. The size of the circles and the color encode the magnitude and the depth of the corresponding events, respectively.

**Figure 4:**
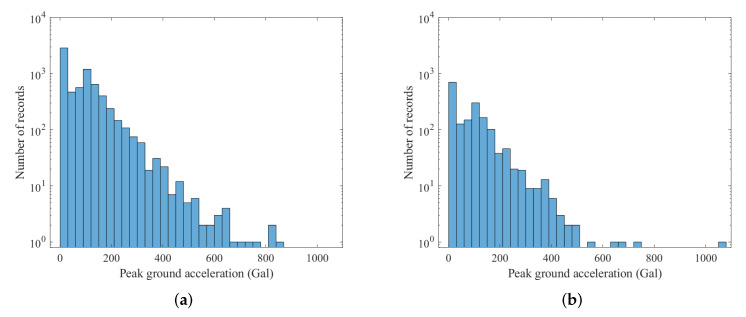
The PGA histograms of the datasets used for training and testing. (**a**) The PGA histogram of the training dataset. (**b**) The PGA histogram of the testing dataset.

The earthquake events were collected from the CWBSN and TSMIP in Taiwan. There are 195 CWBSN stations and 495 TSMIP stations as shown in Figure 2. The datasets consist of 896 events recorded by CWBSN from 2014 to 2019 and 724 events recorded by TSMIP from 1991 to 2019. The events with magnitude greater than 4.0 were chosen for the experiments. The chosen event had focal depths ranging from 0 to 270 km. Figure 3 shows the histograms of the magnitudes, the depths, and the geographical locations of the selected events. The magnitude and depth are encoded by the circle and color, respectively. The PGAs are measured from the maximum amplitude of the three-axis composite wave. The acceleration is sampled at 100 Hz. The same amount of data for the records with a PGA greater than or equal to 80 Gal and those with PGA less than 80 Gal were collected for the purpose of data balance. Finally, there are 6900 acceleration records in the training set and 1720 acceleration records in the testing set. Figure 4 shows the histograms of the PGAs for the records in the training and testing sets.

### 4.2. Prediction Accuracy

Precision, recall, and F1-score are used as the metrics to evaluate the performance [25]. Denote the prediction results as the following four cases, namely true positive (TP), true negative (TN), false positive (FP), and false negative (FN). Precision is the fraction of TP records among all predicted positives. Recall is the fraction of TP records among all actual positives. The measures can be defined as follows:

(5) $$\mathrm{Precision}=\frac{\mathrm{TP}}{\mathrm{TP}+\mathrm{FP}}\;\;\;\mathrm{Recall}=\frac{\mathrm{TP}}{\mathrm{TP}+\mathrm{FN}}\;\;\;F1\text{-}\mathrm{score}=2\times \frac{\mathrm{Precision}\times \mathrm{Recall}}{\mathrm{Precision}+\mathrm{Recall}}.$$

Note that F1-score is the harmonic mean of precision and recall. In general, with a high F1-score, precision and recall must also be high.

The proposed ISMP scheme is compared to the threshold-based scheme proposed in [9]. Similar to our scheme, the threshold-based scheme predicts whether the on-site PGA of the on-going event will be greater than a threshold η using the peak displacement (Pd) in the initial window of the P-wave. In other word, if Pd in the initial window is greater than a pre-selected threshold δ, it predicts the PGA will be greater than η. Otherwise, it predicts the PGA will not be greater than η. For η equal to 80 Gal, the paper suggests Pd threshold δ to be 0.35 cm.

**Figure 5:**
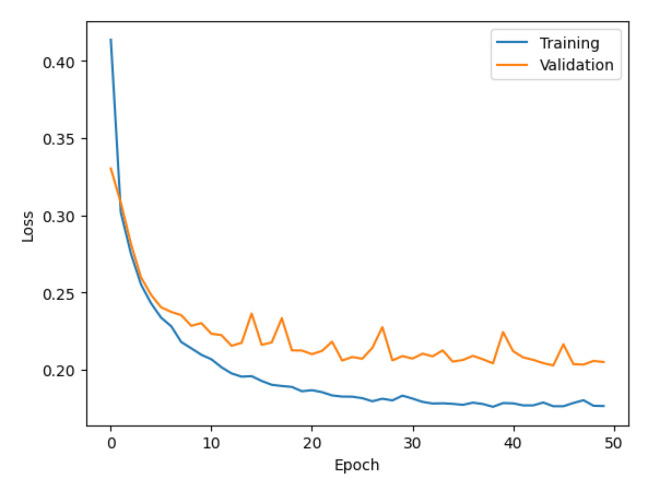
The training and validation process for the model using a three-second time window of the initial P-wave.

In the experiments, five ISMP models were built for using a one- to five-second time window of the initial P-wave to predict the PGA condition. The number of parameters in the five models are 229,250, 331,650, 434,050, 536,450, and 638,850 for one- to five-second time window models, respectively. Figure 5 shows a typical training and validation process for the built model, which used a three-second time window of the initial P-wave as input. The training process converged at about 50 epochs and the loss of the validation was also close to the training results. The inference time is about 0.08 s on an Intel i7 PC. The quick inference time supports the requirement for earthquake early warning.

**Figure 6:**
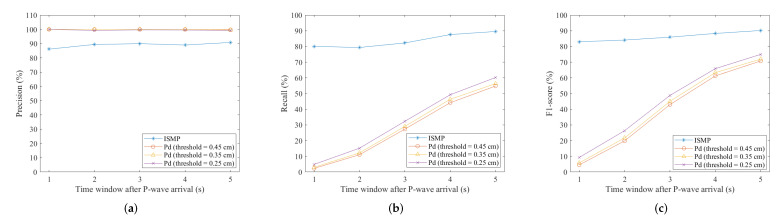
Accuracy measurements. (**a**) Precision. (**b**) Recall. (**c**) F1-score.

Figure 6a,b show the precision and recall of the two methods, respectively. Figure 6a shows that the precision of the Pd scheme is almost 100 percent, while the precision of the ISMP scheme is a little worse in the precision for prediction. A high precision score indicates that the false alarm rate is low. However, in Figure 6b, the recall of the Pd scheme is much lower than that of the ISMP scheme. A low recall score indicates that the missed alarm rate is high. Therefore, the high precision of the Pd scheme results from missing many events with PGA greater than 80 Gal. The kind of errors is not desirable, either, even the precision of the prediction is high. The ISMP scheme has a missed alarm rate of under 20% in all time windows. The ISMP scheme can thus predict correctly for more records with large PGAs than the Pd scheme.

To integrate the two metrics above, the F1-scores for the two schemes are shown in Figure 6c. The ISMP scheme is obviously better than the Pd scheme using any length of the initial time window. In general, the F1-score increases as the time window increases, because the longer the time window, the more information that can be obtained from the input records. From the work in [9], a three-second initial time window is suggested for making the prediction. By using a three-second time window, the F1-score of the ISMP scheme is greater than 85%. The Pd scheme depends only on the value of amplitude and may miss certain useful information from the data. The performance is thus worse than the ISMP scheme. From these results, the ISMP scheme exhibits better performance on issuing alerts and is more appropriate for on-site EEW systems.

### 4.3. Performance on the Sample Cases

Two sample events were used to show the performance of the ISMP and Pd schemes. The Pd threshold for the Pd scheme was set to 0.35 cm. In the experiments, the leading time is also investigated. The leading time is the warning time from the end of the time window to the time of the maximum amplitude of PGA. A longer leading time indicates a longer time available for reaction. Denote $t_{i}^{\prime }$ as the time of the maximum amplitude of the *i*th record, and $t_{i}$ as the time that the alert is triggered. The leading time is defined as $t_{i}^{\prime }-t_{i}$.

**Figure 7:**
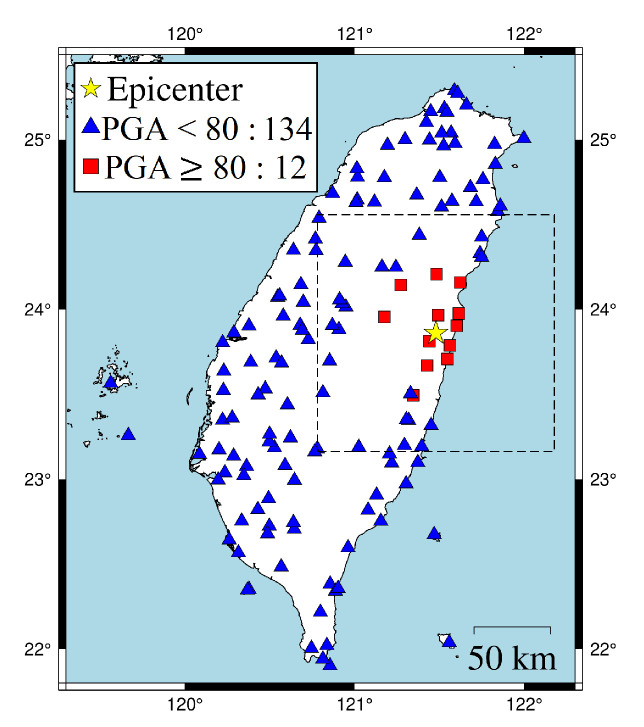
18 April 2021 Hualien earthquake ($M_{L}$ = 6.2, depth = 14.4 km). The dotted box is the area close to the hypocenter and chosen to be investigated in Figure 8.

**Figure 8:**
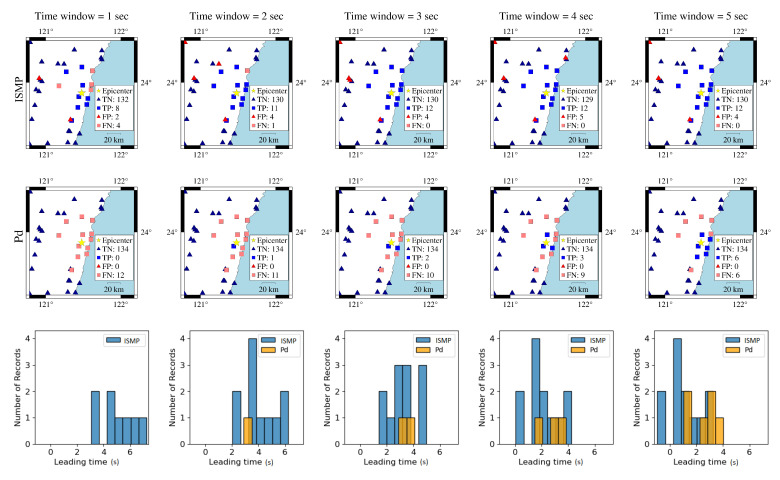
Comparison of ISMP and Pd schemes using different time windows for the 18 April 2021 Hualien event. The first and second rows are the prediction results of the ISMP and Pd schemes, respectively. The third row shows the histogram of the leading time in TP records using the two schemes. The yellow star represents the event epicenter. The triangles are the stations with actual PGA < 80 Gal and the squares are the stations with actual PGA ≥ 80 Gal, where the blue and red colors represent stations that make correct and false predictions, respectively.

In the first case, the 18 April 2021 magnitude 6.2 Hualien earthquake is selected. Figure 7 shows the actual conditions for PGA ≥ 80 Gal and PGA < 80 Gal at the seismographic stations. There are 12 stations with PGA ≥ 80 Gal and 134 stations with PGA < 80 Gal. The blue triangles are the stations with actual PGA < 80 Gal. The red squares are the stations with actual PGA ≥ 80 Gal. To compare the on-site early warning performance, the area close to the epicenter, which is the dashed square in Figure 7, was further investigated in Figure 8. The results show that, using a time window of one second, eight stations with actual PGA ≥ 80 Gal successfully predicted the PGA conditions near the epicenter using ISMP. All correct alarm stations had over three seconds of leading time. However, the Pd scheme produced no correct alarms, and, thus, there was no leading time at any station. Using a time window of three seconds, all the stations with actual PGA ≥ 80 Gal correctly predicted the PGA condition, but the Pd scheme only supplied two correct alarm stations. As the time window increased, the number of correct alarms triggered by the ISMP scheme also increased, but the leading time became relatively shorter. It demonstrates the trade-off between the leading time and the number of correctly predicted stations. The leading time histograms are shown in the third row of Figure 8. For the ISMP scheme, using one- to five-second time windows yields average leading times of 4.97, 4.1, 3.12, 2.12, and 1.12 s, respectively. For EEW systems, the leading time is as important as the prediction accuracy. Therefore, time window selection is vital.

**Figure 9:**
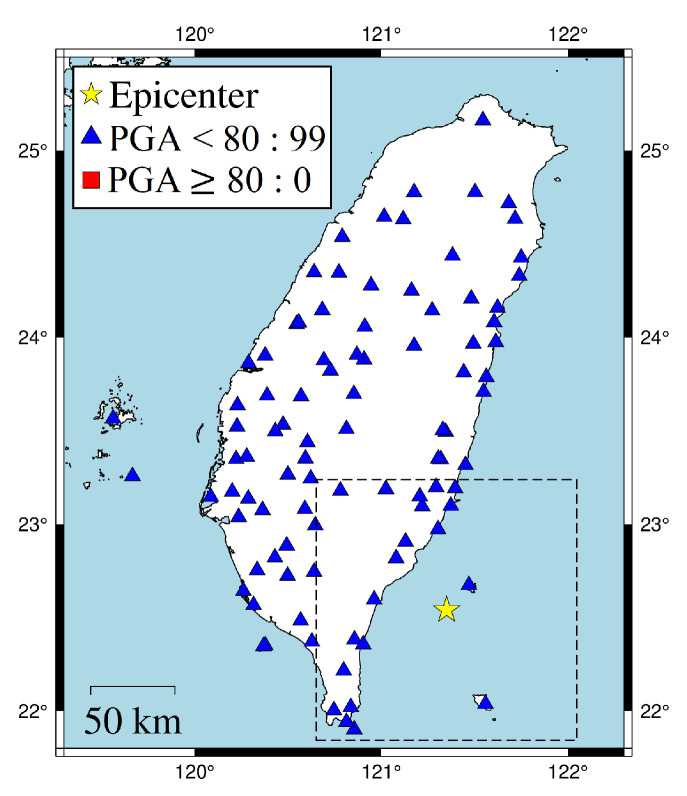
17 January 2021 Taitung earthquake ($M_{L}$ = 5.5, depth = 21.1 km). The dotted box is the area close to the hypocenter and chosen to be investigated in Figure 10.

**Figure 10:**
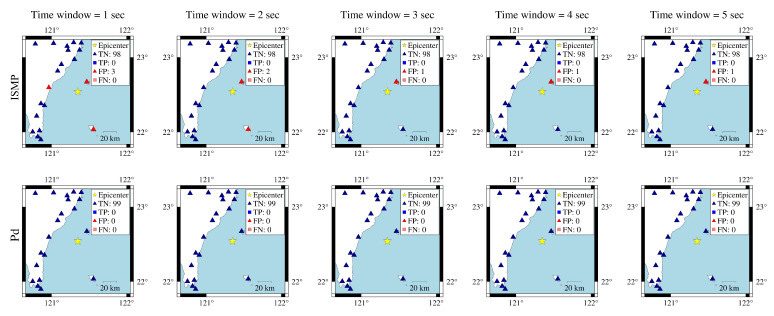
Comparison of ISMP and Pd schemes using different time windows for the 17 January 2021 Taitung event. The first and second rows are the prediction results of the ISMP and Pd schemes, respectively. The yellow star represents the event epicenter. The triangles are the stations with actual PGA < 80 Gal and the squares are the stations with actual PGA ≥ 80 Gal, where the blue and red colors represent stations that make correct and false predictions, respectively.

In the second case, the 17 January 2021 magnitude 5.5 Taitung earthquake is used and shown in Figure 9. The event was out of the island and under the sea. There are no records with PGA ≥ 80 Gal in this case. Figure 10 shows that, as the time window increased from one to three seconds, the number of false alarms triggered by the ISMP scheme decreased. For time windows of three to five seconds, the prediction results of the ISMP scheme were the same. It indicates that a time window of three seconds is sufficient to obtain enough information from the data to make the correct prediction. Only one false alarm was generated, and the PGA at that station was 73.08 GAL which is very close to 80 Gal. The ISMP scheme mistakenly overestimated the potentially destructive force of the seismic wave at this station. However, it is not a harmful result. On the other hand, the Pd scheme makes all correct predictions for the intensity of the ground motion at the stations. Recall that the Pd scheme is better in the precision performance and worse in the recall. In other words, if the Pd scheme uses a high threshold for Pd, it can reduce false alarms but may result in a higher missed alarm rate. Since no station has a PGA greater than 80 GAL in this event, there is no missed alarm for the positive case. Thus, the Pd scheme shows a high precision.

From the evaluations, the ISMP scheme can predict records with large PGA values more successfully and allow for longer leading times than the Pd scheme. Using neural network techniques, the ISMP scheme can capture more key characteristics for predicting potentially destructive waves and may perform better than the traditional methods.

## 5. Conclusions

The proposed intelligent strong motion prediction (ISMP) model is designed to predict early ground motion. ISMP uses a convolutional neural network to effectively extract relevant features from the initial P-waves to predict whether the peak ground acceleration of subsequent waves surpasses 80 Gal. Earthquakes with many strong motion records were selected to train and test these models, and experiments were conducted to evaluate their performance. The ISMP scheme was compared to the threshold-based Pd scheme through the testing set and some sample cases. From the evaluations, the ISMP scheme demonstrates higher F1-scores from the testing set. In the sample cases, the ISMP scheme significantly outperforms the Pd scheme. In the future, deploying the proposed ISMP scheme in some areas that require rapid warnings and evaluating the performance of the scheme in real time will be investigated.

## Data Availability

An implementation of ISMP is available at https://github.com/aszx232k/ISMP (accessed on 23 December 2021).
